# Managing Femoral Artery Pseudoaneurysm Using Snare-Guided Through-and-Through Access With Perclose ProGlide

**DOI:** 10.7759/cureus.77650

**Published:** 2025-01-19

**Authors:** Shojiro Oka, Masaya Fumimoto, Shunjiro Noguchi, Riki Sakano, Shigeshi Kohno

**Affiliations:** 1 Diagnostic Radiology, Kobe City Medical Center General Hospital, Kobe, JPN; 2 Diagnostic Radiology, Kitano Hospital, Osaka, JPN; 3 Gastroenterological Medicine, Kobe City Medical Center General Hospital, Kobe, JPN

**Keywords:** femoral artery pseudoaneurysm, gooseneck snare, perclose, proglide, through-and-through wire technique, vascular closure device

## Abstract

Iatrogenic femoral artery pseudoaneurysm (IFAP) is a common complication following endovascular procedures. Treatment options include manual compression, ultrasound-guided compression, thrombin injection, and surgical repair, each with its own limitations. Recent case reports have described successful IFAP treatment using the Perclose ProGlide/ProStyle (Abbott Vascular, Lake Bluff, USA) suture-mediated closure device by directly puncturing the pseudoaneurysm sac and advancing a guidewire into the native artery. Herein, we present a novel through-and-through wire technique using the same device for treating IFAP. The technique involves advancing a microcatheter into the pseudoaneurysm from the contralateral femoral artery, deploying a snare through it, and then puncturing through the center of the deployed snare to establish through-and-through wire access for Perclose deployment. We successfully treated a 60-year-old woman who developed a femoral artery pseudoaneurysm following angiography. Compared to previously reported direct puncture techniques using Perclose, this approach allows real-time angiographic confirmation of device deployment and may reduce radiation exposure to operators compared with direct puncture techniques. While further experience is needed to determine its optimal role in clinical practice, this technique may serve as an effective alternative in the endovascular treatment of IFAP.

## Introduction

Iatrogenic femoral artery pseudoaneurysm (IFAP) is a common complication after endovascular procedures, with an incidence rate of 2.0-7.7% [1-3]. Traditional management includes manual compression, ultrasound-guided compression, thrombin injection, and surgical repair [4,5].

Manual compression can be effective for smaller pseudoaneurysms but may not be as successful in cases involving large cavities, wide necks, or ongoing anticoagulation [6]. Ultrasound-guided thrombin injection is another minimally invasive option, but it carries the risk of thrombosis, particularly in pseudoaneurysms with short and wide necks [7]. Surgical repair remains the gold standard for complex IFAPs, including those with large sizes, rapid expansion, infection, or associated neurovascular compromise. However, surgery is associated with longer hospital stays, increased risk of wound infection, and higher morbidity [8].

Successful treatment of IFAP using a percutaneous suture-mediated vascular closure system, Perclose ProGlide/ProStyle (Abbott Vascular, Lake Bluff, USA), has been recently reported [9,10]. This technique involves directly puncturing the pseudoaneurysm sac under ultrasound guidance, advancing a guidewire through the neck into the native artery, and deploying a closure device to seal the defect. However, accessing the native artery can be challenging when the pseudoaneurysm has an irregular morphology or a narrow or tortuous tract. Here, we present a novel technique that involves approaching a femoral artery pseudoaneurysm from the native artery side using Perclose in a woman in her 60s and comparing it with traditional IFAP management methods. This approach offers an additional strategy for endovascular treatment of IFAP.

## Case presentation

**Figure 1:**
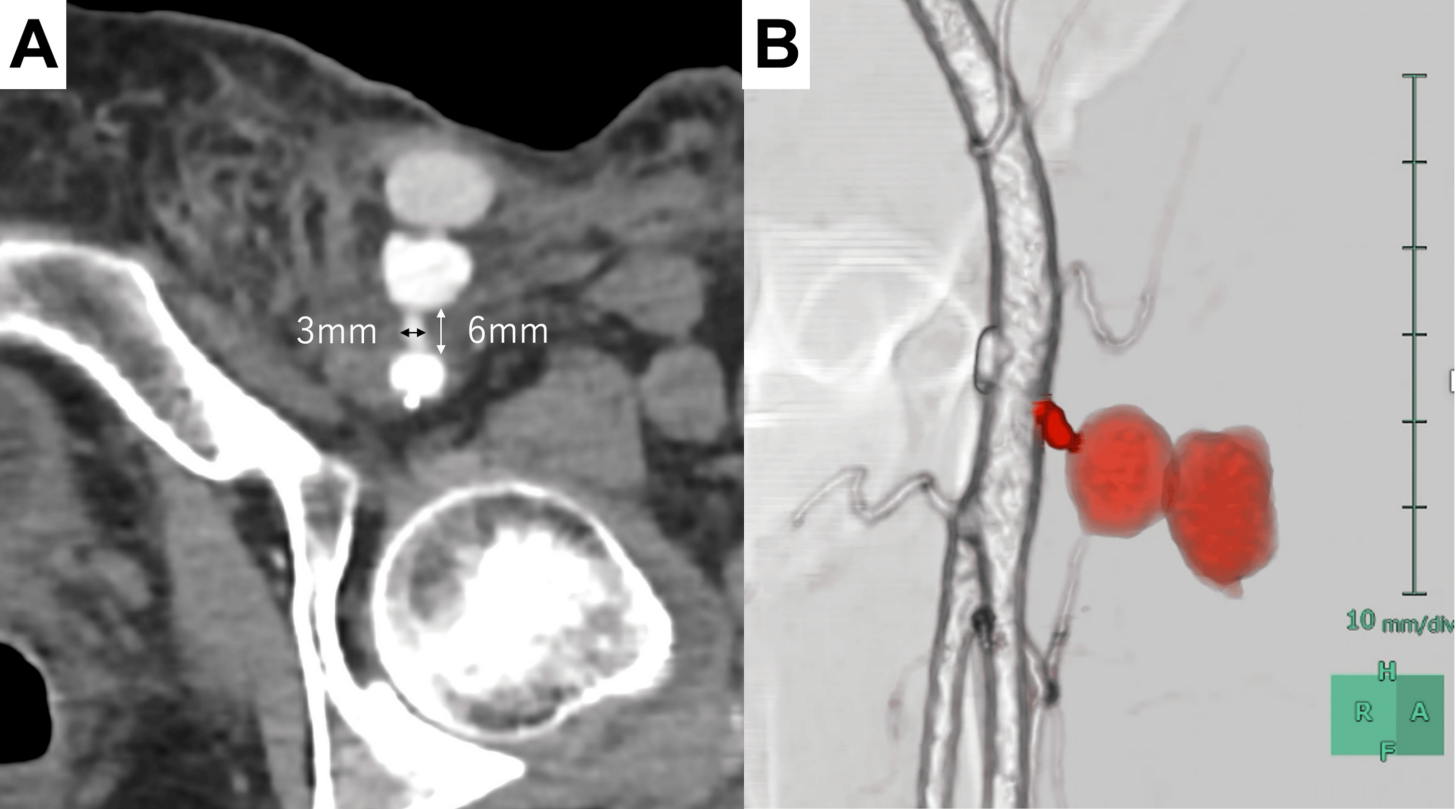
Contrast-enhanced computed tomography images of the left femoral artery pseudoaneurysm A: Axial image; B: Volume-rendering image created using SYNAPSE VINCENT (Fujifilm, Tokyo, Japan). The red color indicates the tract and pseudoaneurysm, and the white color shows the left femoral artery. Colors were added during postprocessing for better visualization of the anatomical structures.

A 60-year-old woman with no significant medical history presented to our hospital with abdominal pain. Contrast-enhanced computed tomography (CT) revealed retroperitoneal hemorrhage, and she underwent angiography for a suspected pancreaticoduodenal artery aneurysm via a 5 Fr sheath through the left common femoral artery. On the day after the procedure, the patient developed groin swelling, and ultrasonography revealed a pseudoaneurysm. The patient did not take any anticoagulation or antiplatelet medications. Laboratory tests showed a decreased hemoglobin level (8.1 g/dL) but normal coagulation parameters. Despite additional compression attempts, effective hemostasis could not be achieved because of extensive hematoma. Contrast-enhanced CT on postprocedural day 2 confirmed the presence of a left femoral artery pseudoaneurysm with a tract length of 6 mm and diameter of 3 mm (Figure 1). Although surgical repair was considered, we first attempted endovascular treatment because it is less invasive.

**Figure 2:**
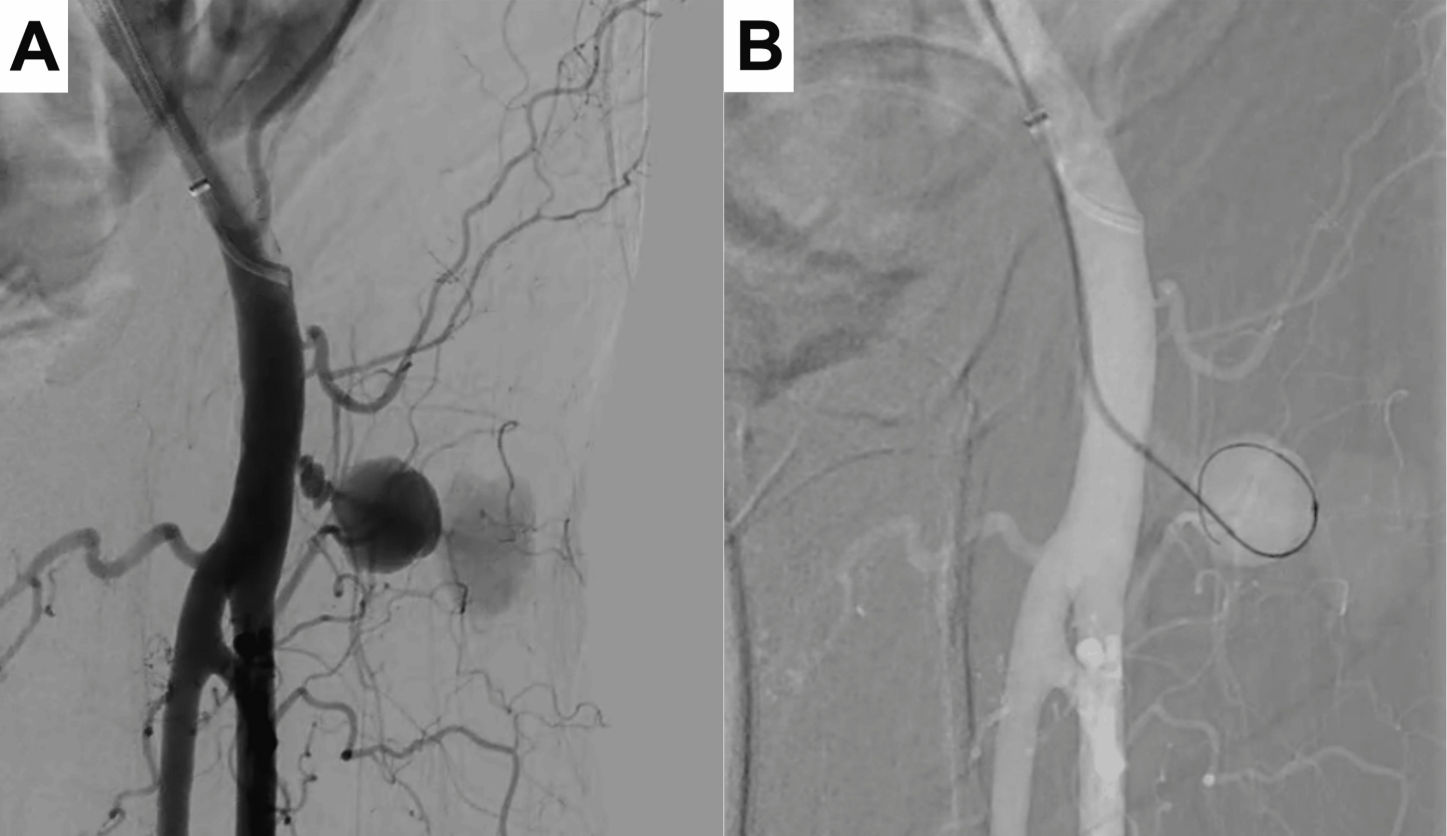
Angiographic images during the procedure A: Initial angiogram showing the pseudoaneurysm from the left common femoral artery; B: Microcatheter system positioned within the pseudoaneurysm

A 6 Fr destination guiding sheath (Terumo, Tokyo, Japan) was placed in the right common femoral artery. The initial angiography revealed a pseudoaneurysm originating from the left common femoral artery (Figure 2A). We then advanced a 4 Fr Cobra catheter (Medikit, Tokyo, Japan), followed by a 2.9 Fr Swift NINJA catheter (SB KAWASUMI, Tokyo, Japan) and a 1.6 Fr Carnelian MARVEL S catheter (Tokai Medical Products, Aichi, Japan) into the pseudoaneurysm via the tract (Figure 2B) using a CHIKAI V guidewire (Asahi Intecc, Aichi, Japan).

A 7-mm Gooseneck snare (Medtronic, Minneapolis, MN, USA) was deployed using the Swift NINJA catheter. Under fluoroscopic guidance, an 18-gauge needle was advanced percutaneously, targeting the center of the deployed snare (Video 1). After confirming that the needle passed through the snare loop, a 0.035-inch guidewire was advanced through the needle. The wire was then captured using a snare and pulled through to create a through-and-through wire access.

**Video 1:** Fluoroscopic-guided snare-targeted needle puncture

**Figure 3:**
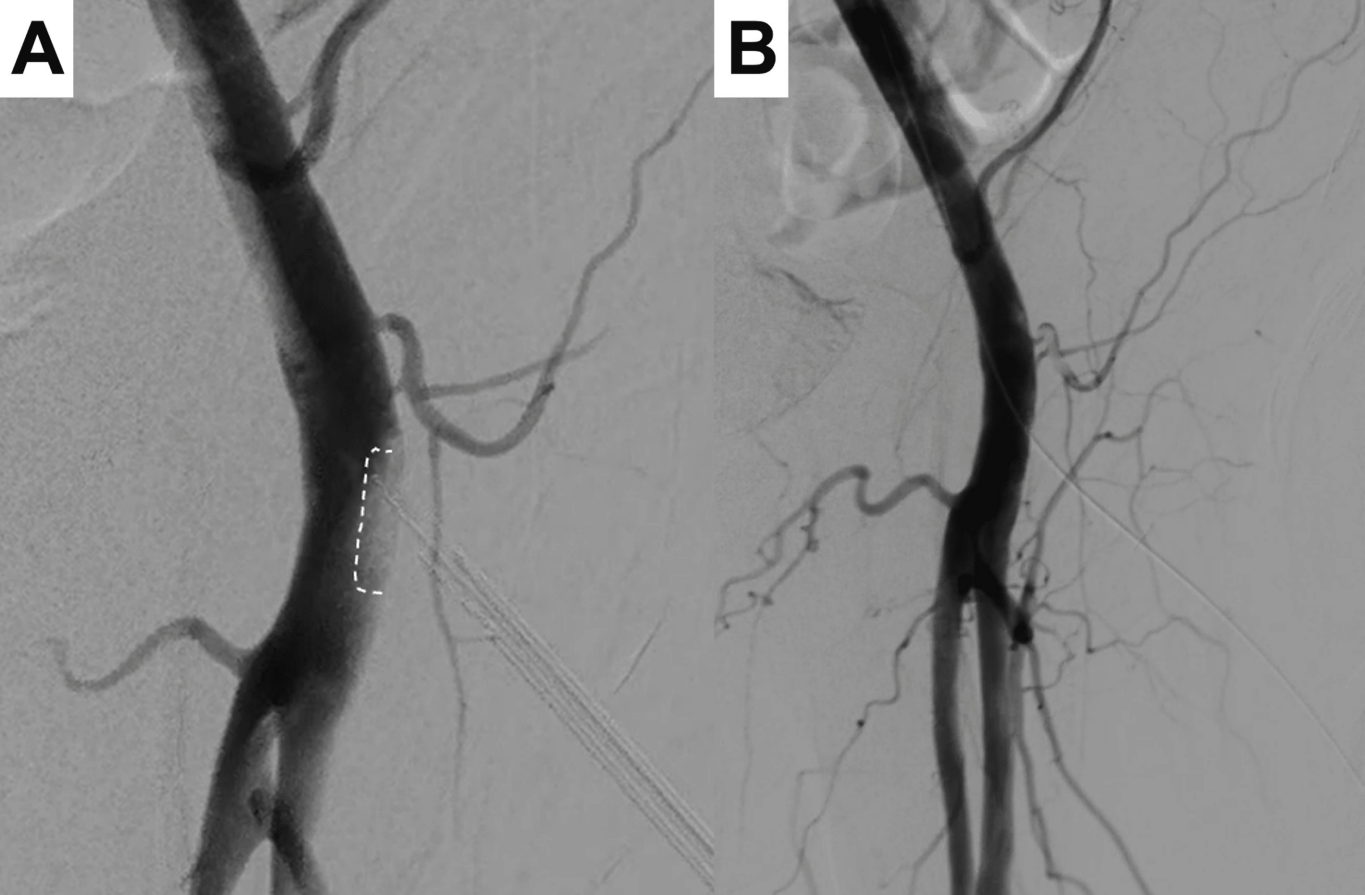
Angiographic images after Perclose deployment A: Angiogram showing the Perclose footplate in proper position (dotted line); B: Final angiogram demonstrating complete pseudoaneurysm closure with preserved arterial flow

Perclose was advanced over the through-and-through wire from the left femoral puncture site. The device was deployed as per standard techniques to achieve immediate hemostasis. Angiography performed using the Perclose footplate confirmed secure apposition to the vessel’s anterior wall, ensuring effective closure (Figure 3A). Postprocedure angiography confirmed the complete closure of the pseudoaneurysm while preserving femoral artery flow (Figure 3B). The patient’s postoperative recovery was uneventful without any evidence of rebleeding.

**Figure 4:**
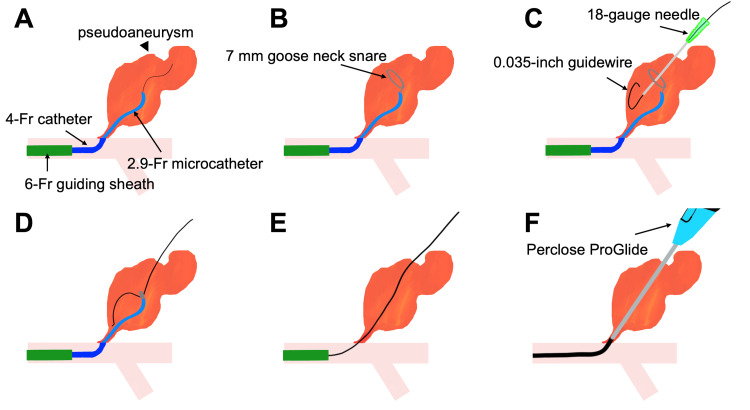
Schematic diagram illustrating the through-and-through wire technique for pseudoaneurysm closure using Perclose Credit: Image created by the authors

A schematic representation of the procedure is shown in Figure 4.

.

## Discussion

Suture-mediated closure systems are commonly used to achieve hemostasis after endovascular procedures. The primary mechanism involves deploying sutures to close arterial puncture sites, with reported technical success rates of 91-100% in various settings [3]. This device is particularly valuable for procedures requiring large-bore access, such as endovascular aortic aneurysm repair and transfemoral aortic valve replacement, where traditional manual compression may be insufficient [11-13]. The device is effective and safe for selected patients with peripheral vascular disease undergoing angiography, including those on anticoagulation therapy [14]. This device shows promise as a minimally invasive treatment for pseudoaneurysm repair, with several case reports describing successful outcomes using direct puncture techniques [9,10]. Another case report described the successful closure of a brachial artery pseudoaneurysm with Perclose, with the procedure involving initial radial artery access and angiographic guidance [15].

Our modified through-and-through technique offers several advantages. First, it serves as an alternative approach when direct puncture fails to provide access to the native artery. Even in cases where pseudoaneurysm selection is difficult, the established arterial access allows an immediate transition to balloon-occlusion thrombin injection as a backup strategy. Second, confirmation of proper Perclose footplate deployment does not rely entirely on the trickling of blood from the marker lumen, as this can happen even while the device is in an extravascular position within the perfused sac of the pseudoaneurysm. This technique allows real-time angiographic confirmation of footplate deployment, potentially reducing the risk of deployment failure. Third, this approach may reduce direct radiation exposure to the operator's hands compared with direct puncture techniques. Previous case reports have shown operators' hands appearing in fluoroscopic images during direct puncture procedures [10,15], indicating potential radiation exposure concerns.

However, the technique has several limitations. First, the use of additional devices, such as microcatheters and snares, increases procedural costs compared with other approaches. Second, the technique requires contralateral femoral artery access, which adds another puncture site and potential complications, particularly in patients with coagulopathy. Third, the technique cannot be applied when the tract diameter is too large to allow proper anchoring of the Perclose footplate against the vessel wall. Fourth, in this case, direct puncture or thrombin injection might have been successful. Finally, it should be noted that using the Perclose device for pseudoaneurysm closure represents an off-label application.

## Conclusions

Although conventional approaches, such as the direct puncture technique and thrombin injection, remain viable, our modified technique may be a valuable and effective alternative for treating femoral pseudoaneurysms. This technique expands the treatment options available for managing IFAPs and has the potential to improve patient outcomes by offering a minimally invasive alternative to surgery in selected cases. More cases are required to establish its optimal role in clinical practice.
